# Macular Hole Closure Between Two Tamponades: A Case Report

**DOI:** 10.7759/cureus.93609

**Published:** 2025-09-30

**Authors:** Rituraj P Videkar, Gangaprasad Amula, Navneet Mehrotra

**Affiliations:** 1 Vitreoretinal Surgery, Fakeeh University Hospital, Dubai, ARE; 2 Ophthalmology, Al Ahli Hospital, Doha, QAT; 3 Retina, Retina Foundation and Retina Care, Ahmedabad, IND

**Keywords:** full-thickness macular hole, pars plana vitrectomy, per fluorocarbon liquid, retinal detachment (rd), silicon oil

## Abstract

In this case report, we discuss the closure of a macular hole over a perfluorocarbon liquid (PFCL) globule in a silicone oil-filled eye. PFCL is an important adjunct in the armamentarium of a vitreoretinal surgeon. The use of PFCL has changed the management of various retinal pathologies. PFCL has increased the success rates of vitrectomies for retinal detachment associated with giant retinal tear and proliferative vitreoretinopathy. Recent literature has reported the use of PFCL in the management of macular hole with or without concurrent retinal detachment. A 60-year-old woman presented with retinal detachment with macular hole in her right eye. On examination, her vision in the right eye was hand movement close to face (HMCF). The intraocular pressure was 14 mmHg. Anterior segment examination was suggestive of pseudophakia, whereas posterior segment examination revealed a total retinal detachment with a macular hole and multiple retinal breaks. The patient underwent right eye vitrectomy with internal limiting membrane (ILM) peeling with endolaser to the retinal break and silicone oil tamponade (5000 centistokes viscosity, Densiron). Intraoperatively, the ILM peeling of the macula was performed using the countertraction effect of PFCL. Her vision on postoperative day 1 was suggestive of 6/60 on Snellen visual acuity chart. The intraocular pressure in the right eye was 12 mmHg. The anterior segment was normal, and the posterior segment was suggestive of an attached retina with a closed macular hole. On careful examination, the presence of subfoveal PFCL was noted, indicating closure of the macular hole over a PFCL globule. PFCL is helpful in vitreoretinal surgery. It has high specific gravity with low viscosity. It is used to stabilize the retina during the membrane peeling maneuvers in vitreoretinal surgery. ILM peeling around the macular hole with coexistent retinal detachment is performed by placing the PFCL globule on the macula. After the ILM peeling, the PFCL globule is carefully removed. However, during the ILM peeling step, the PFCL globule can sneak in the subretinal space through the macular hole. This occurs due to preexisting retinal detachment and the tractional forces of the ILM. The residual PFCL globule can be missed, as in our case, where the macular hole was found to be closed over the PFCL globule. The subfoveal location of the PFCL globule is associated with toxicity to retinal photoreceptors and is associated with poor prognosis. Meticulous removal of PFCL is necessary during the vitreoretinal surgery. Triamcinolone acetonide adsorbs onto PFCL globule and aid in its removal. The use of PFCL globule as an adjunct during ILM peeling in vitreoretinal surgery for macular hole with concurrent retinal detachment can be associated with subfoveal migration of PFCL and closure of the macular hole over the PFCL globule.

## Introduction

Perfluorocarbon liquid (PFCL) has revolutionized modern-day vitrectomy procedures. PFCL has low viscosity and high specific gravity. Due to this inherent property, it is used as a tool to stabilize the retina during vitrectomy. It is also used as a cushion to prevent iatrogenic retinal trauma during vitrectomies for intraocular foreign body removal and nucleus drop removal. Similar to silicone oil, PFCL can be used as a temporary retinal tamponade. PFCL has changed the management pattern of various retinal pathologies, positively impacting the success rate of vitrectomies for retinal detachment with giant retinal tear, proliferative vitreoretinopathy, etc. However, the use of PFCL has its own pitfalls. Migration of PFCL into the anterior chamber and subretinal location has been reported in the literature. The subfoveal location of PFCL warrants removal, as it is associated with retinal toxicity. Its removal can be performed by direct aspiration, therapeutic macular hole creation, or by inducing retinal detachment [1-5]. In this case report, we discuss the closure of a macular hole over the PFCL globule due to its subfoveal migration during internal limiting membrane (ILM) peeling in a case of macular hole with concurrent retinal detachment. This is one of the rare case reports, as closure of a macular hole over the PFCL globule in a silicone-filled globe has not been reported in the literature.

## Case presentation

**Figure 1:**
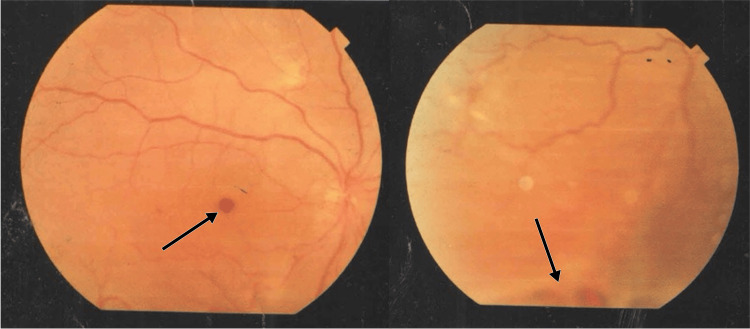
Right eye fundus photo suggestive of retinal detachment with a macular hole. The marking in the first photo highlights the macula hole, whereas the marking in the second photo highlights the presence of retinal detachment with a retinal break at the 6 o'clock position.

A 60-year-old woman presented with poor vision in the right eye. She gave a history of undergoing cataract extraction in both eyes a few years ago. She accidentally noticed poor vision in the right eye. On examination, the vision in the right eye was HMCF, whereas in the left eye measured 6/6 on Snellen visual acuity chart. The intraocular pressure in both eye was 14 mmHg. The anterior segment examination in both eyes was unremarkable with the presence of well-centered intraocular lens. The fundus examination in the right eye revealed the presence of complete posterior vitreous detachment with total retinal detachment and a macular hole. Indentation examination of the retina revealed the retinal break at 12, 3, and 6 o'clock positions. The left eye fundus examination was normal. The patient underwent fundus photography (Figure 1) and was taken up for right eye vitrectomy, ILM peeling, endolaser to the retinal breaks along with silicone oil (5000 centistoke viscosity, Densiron) the next day.

**Figure 2:**
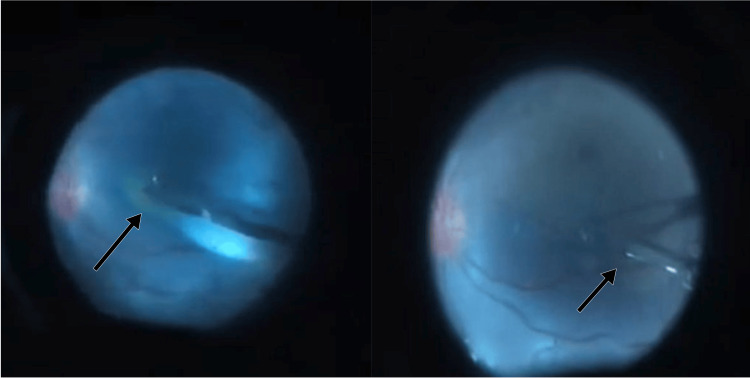
ILM peeling under the PFCL globule. The arrow in the first photo highlights the initiation of the ILM peel under the PFCL globule, whereas the arrow in the second photo highlights the completion of the ILM peel under PFCL. The PFCL globule provides countertraction during ILM peel in a detached retina, thus providing a working platform. This is the surgeon's view (inverted view) of the operative field of the right eye where the surgeon is sitting at the head end of the patient. ILM: internal limiting membrane; PFCL: perfluorocarbon liquid.

During surgery, after an uneventful vitrectomy, ILM peeling was undertaken. The ILM was stained with brilliant blue dye, which was followed by peeling maneuver. For performing ILM peeling, PFCL was injected over the macula to stabilize the retina. The ILM peel was then performed under the PFCL in order to close the macular hole (Figure 2).

**Figure 3:**
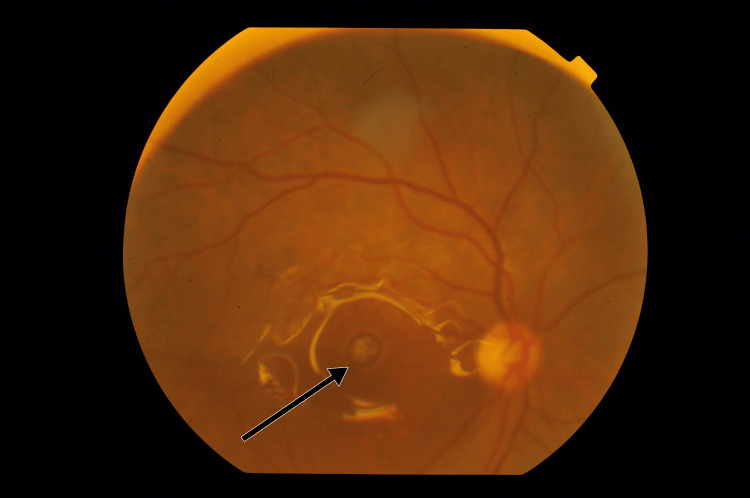
Subfoveal PFCL with a closed macular hole in a silicone oil-filled eye with an attached retina. The arrow highlights the pearly appearance of PFCL, which is in subfoveal location. The shiny reflex around the PFCL globule is due to silicone oil. The retina is attached. Macular hole has closed over the PFCL globule, thus trapping the PFCL globule in subfoveal location. This is macular hole closure between two tamponades. PFCL: perfluorocarbon liquid.

**Figure 4:**
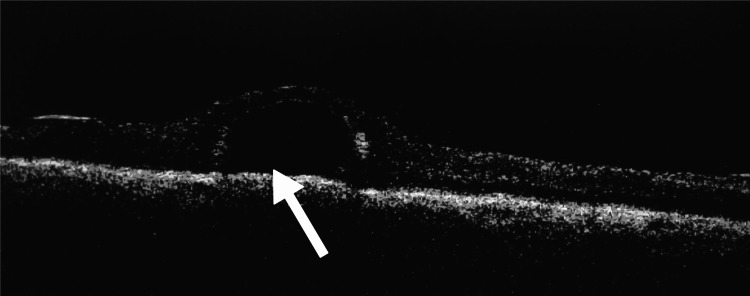
OCT line scan highlighting the "omega sign" of subfoveal PFCL. The arrow highlights an optically clear hyporeflective space in the subretinal location. The steep angle of contact and between the inner retina and retinal pigment epithelium along with thinned out retina over the hyporeflective space indicates the presence of PFCL in the subfoveal location. OCT: optical coherence tomography; PFCL: perfluorocarbon liquid.

This was later followed by fluid exchange and endolaser to the retinal breaks. The surgery was then concluded by using Densiron (silicone oil with a viscosity of 5000 centistokes) as a retinal tamponade. Postoperatively, the patient was advised to sleep in a supine position so as to achieve good retinal tamponade. After the surgery, vision in the right eye improved to 6/60 on the Snellen visual acuity chart, and the intraocular pressure in right eye was 12 mmHg. Anterior segment examination showed pseudophakia, and fundus examination revealed a silicone oil-filled eye with an attached retina and a closed macular hole. On careful examination, the presence of subfoveal PFCL was noted, indicating closure of the macular hole over the PFCL globule. The patient underwent fundus photography (Figure 3) and optical coherence tomography (OCT) examination to confirm the findings (Figure 4).

The fundus photograph showed evidence of an attached retina with subfoveal PFCL. The OCT line scan taken at 180° showed hyporeflective space in the subretinal area with a steep angle of separation between the inner retina and retinal pigment epithelium with thinned out overlying retina. This was suggestive of subfoveal PFCL and closure of macular hole over it. The patient was explained about the complication and the need for the removal of subfoveal PFCL globule. The patient subsequently underwent silicone oil removal after four weeks.

## Discussion

On account of its high specific gravity, PFCL can be used to provide countertraction during membrane peel. PFCL is widely used in cases of retinal detachment with proliferative vitreoretinopathy. ILM peel has drastically improved the success rates in macular hole surgeries. ILM can be easily peeled off the macula in cases of attached retina. However, the same maneuver becomes challenging in cases of macular hole with concurrent retinal detachment. PFCL can act as a stabilizer to provide countertraction, thus providing a working platform in such situation. Incomplete removal of PFCL is associated with ocular toxicity, so PFCL should be dealt with caution. In case of macular hole with concurrent retinal detachment, regrasping the ILM during membrane peel may be difficult as the PFCL bubble will flatten the flap as soon as it is dropped. There have been recent reports where PFCL marble is injected over the macular hole, followed by ILM peeling around it under infusion fluid during vitrectomy [6]. The use of PFCL has its own drawbacks, with the retention of PFCL being one of them. Retention of PFCL after vitrectomy is toxic to ocular tissues. Its retention in anterior chamber is toxic to the corneal endothelium, whereas in the subretinal space it is toxic to retinal photoreceptors. Subretinal PFCL is known to occur in cases associated with extensive retinectomies [7]. Subretinal retention of PFCL is rare occurrence with an incidence in the range of 0.9 to 11.1% [7,8]. The retention of PFCL in subfoveal space becomes a cause of concern as PFCL is known to damage the outer segment of photoreceptors in animal models, along with macrophage-predominated inflammation in the area of retention [9,10]. In such situation, PFCL is known to be found in intracellular vacuoles [10]. Subfoveal PFCL induces a scotoma, which has been documented on microperimetry by a study that also reported subsequent improvement in visual field after PFCL removal [11]. The toxic effect of PFCL depends on the location, duration of contact, and size of the PFCL globule [12]. There have been reports of spontaneous resolution, macular hole formation, and release of retained subfoveal PFCL [13-15]. Timely detection of PFCL globule is the most important step in its removal and preventing its retention.

Key points while using PFCL

**Figure 5:**
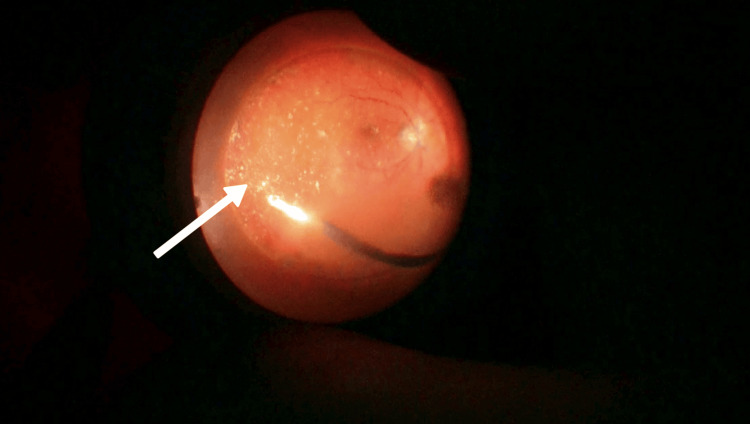
Adsorption of triamcinolone crystals on a PFCL globule placed on the posterior pole of the retina (demonstrative intraoperative image from our center, not from the reported patient). Triamcinolone acetonide crystals 1 mg/0.1 ml, used during vitrectomy for highlighting vitreous strands, can adsorb onto PFCL globule, which gives a distinct look to PFCL globule. Inability to detect PFCL globule during fluid-air exchange is an important reason for the postoperative retention of PFCL in the vitreous cavity. PFCL highlighted by triamcinolone acetonide crystals becomes easily detectable, reducing the chances of postoperative retention. PFCL: perfluorocarbon liquid.

Disruption of a large PFCL globule into smaller PFCL globules occurs due to fluid currents during vitrectomy. This disruption into smaller globules is known as fish egging. This can also take place due to rapid injection of the PFCL in the vitreous cavity leaking to disruption of the large PFCL globule into multiple smaller globules. The use of a dual bore cannula and injecting within the center of globule are some of the ways to avoid fish egging. Fish egging is one of the key reasons of postoperative retention of PFCL. The vitreous cavity should be thoroughly checked after the removal of the PFCL globule to look for any retained globules. Some authors also suggest to inject some saline, which can help in the easy detection of retained globule because saline and PFCL globule are immiscible and will show an interface [16]. The same principle is followed during direct PFCL-silicone oil exchange method, which can also be used to reduce the chances of PFCL retention. Triamcinolone acetonide is widely used in vitrectomy surgery to stain the posterior vitreous cortex. The particulate nature of triamcinolone acetonide helps it to stain the vitreous fibers, making them visually distinct [17]. This property of triamcinolone acetonide can be utilized to highlight the PFCL globule as well. We recommend the use of 0.1 ml of 1 mg/0.1 ml triamcinolone acetonide to detect retained PFCL globule. The triamcinolone acetonide crystals adsorb onto the PFCL globule, helping its easier detection (Figure 5).

There have been recent reports of PFCL globule being used for forced intraoperative closure of macular hole [18]. To the best of our knowledge, this is the first case report of closure of macular hole over a PFCL globule in a silicone-filled eye. In addition to this, our case is distinct due to the presence of a macular hole with concurrent retinal detachment. In our case, we believe that fish egging led to small PFCL globules, one of which made its way to the subretinal space during the ILM peeling maneuver. The shiny light reflexes in the operative field during fluid-air exchange prevented the timely detection of this PFCL globule. Supine position advised during postoperative period further helped to gravitate the PFCL globule toward the base of the macular hole. Densiron (5000 centistokes silicone oil), which was used as a retinal tamponade agent, is heavier than water and gravitates towards the posterior pole. This created a dehydrated state of the macular hole, forcing out any subretinal fluid and leading to the prompt closure of the macular hole over the PFCL globule. 

## Conclusions

PFCL is an effective tool during vitreoretinal surgery. In macular hole with concurrent retinal detachment, PFCL can aid ILM peeling but carries the risk of subfoveal migration. Complete removal of PFCL after vitrectomy is mandatory. Retained PFCL is associated with ocular toxicity. Residual PFCL globule can be hard to detect due to shiny light reflexes in the operative field. Staining PFCL globule with triamcinolone acetonide can help in the detection of PFCL globule, thus aiding complete removal and reducing the chances of retention. Incomplete removal of PFCL at the end of vitrectomy for macular hole with concurrent retinal detachment can be associated with its subfoveal migration. In such scenario, macular hole can close over the PFCL in a silicone oil-filled eye.
